# Patient Engagement in Patient Portals in Appalachia v. Surrounding U.S. Census Regions: An Analysis of HINTS (Health Information National Trends Survey) Data, 2017–2020

**DOI:** 10.13023/jah.0502.05

**Published:** 2023-08-01

**Authors:** Heather Lea Tudor, Rick Ingram, Sarah Wackerbarth

**Affiliations:** Eastern Kentucky University, heathertudor08@gmail.com; University of Kentucky, Richard.Ingram@uky.edu; sbwack0@uky.edu

**Keywords:** Appalachia, patient engagement, patient portals

## Abstract

**Introduction:**

Those living in the Appalachian Region face a greater number of significant health disparities than residents of other areas of the U.S. Patient portals can decrease disparities, increase health literacy, and improve health outcomes.

**Purpose:**

This study explores if those living in the Appalachian Region are offered access to and use their patient portals differently than those in the surrounding U.S. Census regions. Additionally, the study aims to determine if there was a difference in reported reasons for the non-use of patient portals.

**Methods:**

A secondary analysis was completed using data from the National Cancer Institute's Health Information National Trends Survey (HINTS) (2017–2020), a nationally representative survey. Descriptive statistics and chi-square tests were used to determine differences in patient portal use between regions.

**Results:**

There was no statistically significant difference between the Appalachian and surrounding U.S. Census regions in being offered access to patient portals. However, there was a statistically significant difference (non-weighted) between regions in the use of patient portals. Common reasons for the non-use of patient portals were a preference to speak directly to the provider and the lack of perceived need to use the portal.

**Implications:**

Providers in the Appalachian Region should be aware of the non-use of patient portals. Moreover, understanding the reported reasons for non-use may help providers tailor educational materials to increase patient portal use.

## INTRODUCTION

In 2019, the U.S. spent $3.8 trillion on health care, which accounted for 17.7% of the gross domestic product (GDP).^1^,^2^ Chronic disease was the top contributor (90%) to U.S. healthcare spending, and it affects more than 60% of the U.S. population.^3^,^4^

While the U.S. as a whole is negatively affected by chronic disease, some regions are disproportionally affected. One such area is the Appalachian Region.^5^ Several factors drive these increased rates: low levels of education and health literacy, lack of access to providers, low income, and lack of resources.^5^,^6^

Research has shown that engaging patients in their care can improve health outcomes.^7^ When patients are engaged, they can better manage their disease, thus helping reduce costs. Patient portals are one tool healthcare providers can use to help engage patients in their care.^8^,^9^ A patient portal is an online tool that allows patients to access their health information, make or cancel appointments, receive appointment reminders, and get education about important health issues.

### Use of Patient Portals

The most recent data from the 2017 Health Information National Trends Survey (HINTS) shows that 60% of insured adults are offered access to their patient portals.^10^ Still, only 37% of adults report using their portal.^10^ Many demographic factors affect the use of patient portals, such as age, sex, race, education, and health insurance status.

Several studies show that most portal users are aged between 41 and 65 years.^10^,^12^ Additionally, women are more likely than men to be offered access to portals (48.4% v. 39.4% [males]), be encouraged to use them (36.5% v. 29.5% [males]), and actually engage in portal use (30.2% v. 23.0% [males]).^9^,^10^,^12^ Race is another factor that may impact portal use, with one study finding that non-Hispanic whites were more likely to use portals.^12^ However, other studies have shown that race and ethnicity were not associated with portal use.^10^,^11^ Interestingly, patients who are non-Hispanic white are offered access to their portals five times more often than those who are non-Hispanic black (68.9% v. 13.3%).^10^ Hispanics are less likely than other ethnicities to be offered access to their patient portals.^10^

Education also plays a role in patient portal access. Patients with a college education (or higher) are twice as likely to report being encouraged to use their portal than those with a high school education (58.9% vs. 37.8%).^11^,^12^ Those with less than a high school education were even less likely to be encouraged to use their portals (27.9%) and less likely to access their portal.^11^

Lastly, differences in health insurance coverage can also influence patient portal use. Patients with health insurance were more likely to access their records than patients who did not have health insurance (28.1% v. 11.9%, respectively).^11^ Additionally, patients with private insurance were more likely to use their portals,^12^ with those on Medicaid and Medicare being four times less likely to do so, according to self-report data on portal engagement (11.1–15.8% v. 71.8%).^10^

There are other reasons, in addition to demographic factors, why patients may not use a portal. The "digital divide" is a disparity associated with the uneven distribution of access to and use of information technologies, which can affect rural areas.^13^,^14^ However, Otokiti et al.^14^ make the case that patients located in rural areas who are motivated to use health information technology and have access to the internet may not be as affected by the digital divide as they once were. Patients with low education levels are also affected by the digital divide.^14^ People with lower education levels are less like to own a personal computer and have access to the internet.^13^ Moreover, there is an association between low education levels and low health literacy.^15^ Studies have shown that patients with low health literacy are less likely to engage in patient portal use.^12^ Demographic representation of those living in the Appalachian Region mirrors these factors that can affect use of patient portals, such as increased age, education, health insurance, low health literacy, and the digital divide.^5^

Despite these barriers to digital engagement in the Appalachian Region, patient portal use brings several potential benefits that could make it a worthwhile pursuit.^8^,^9^,^11^,^16^ Studies show that patient portal use can increase patient– provider communication, increase quality of care, improve disease outcomes, and increase the patient's ability to manage their chronic conditions.^8^ One study showed that patients who received a message via their portal were more likely to receive an influenza vaccination than the usual care group (n = 39, 137; OR 1.07).^17^ Additionally, patients who received cancer screening reminders were more likely to receive screenings than those who were not reminded.^18^,^19^ Lastly, diabetic patients who began using their patient portals saw an increase in medication adherence and a decrease in their hemoglobin A1C.^13^

Adding to this knowledge, the present study examines the rate at which people living in the Appalachian Region are offered access to and use their patient portals compared to residents of the surrounding U.S. census regions. It additionally evaluates the barriers to patient use that people in Appalachia report facing compared to those in other areas.

## METHODS

### Survey Data

Secondary data analysis was conducted using the National Cancer Institute (NCI) Health Information National Trends Survey (HINTS) to answer this study's central questions. Data from HINTS 5 Cycles 1–4 (2017–2020)^1^ were merged across iterations to increase the sample size.

### Population and Sample

**Figure 1:**
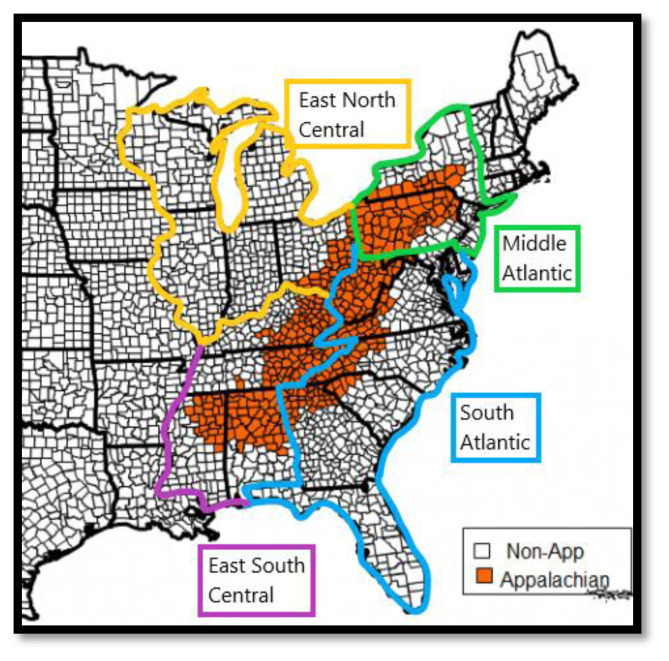
Appalachian Region v. surrounding U.S. Census regions

Subjects included in the sample were all respondents of the NCI's HINTS 5 Cycle 1–4 (2017–2020) who lived in Appalachia or in the surrounding U.S. Census Regions (Figure 1), as defined by the HINTS Data set. HINTS defines Appalachia via the Appalachian Regional Commission’s (ARC) definition and the U.S. Census Regions using the nine Census divisions (East North Central, East South Central, South Atlantic, and Middle Atlantic). The sample included n = 960 from the Appalachian Region and n = 7,388 from the surrounding U.S. Census regions. There were no exclusions based on age, race, ethnicity, income, or other demographic factors in this population.

### Data Analysis

Using SAS 9.4, descriptive and inferential statistics were calculated. Bivariate analyses were conducted using a chi-square weighted jackknife replication variance estimation. Due to variations between regions in race and education level, education and race were controlled to look at differences in patient portal use. The Appalachian Region was also analyzed independently from the surrounding U.S. Census region.

### Measures

The following questions from the dataset were analyzed to achieve the aims of this study:[Table t3-jah-5-2-50]

**Table t3-jah-5-2-50:** 

HINTS question	Response options
Does your health care provider maintain medical records in an electronic format?	Yes, No, Unsure
Have you ever been offered online access to your medical records by your health care provider?	Yes, No, Unsure
How many times did you access your online medical record in the last 12 months?	Yes, No, Unsure
Why have you not accessed your medical records online?	Responses Vary

## RESULTS

### Characteristics of Sample Population

The characteristics of the sample population are shown in Table 1 (next page).

### Provider Maintained EHR

Both regions (Appalachian Region and surrounding U.S. Census region) report a relatively high proportion of providers who use electronic medical records (EMR), at 78–81%. Missing variables were excluded from the analysis. Results showed a statistically significant difference in the number of providers who maintained an EMR between the two regions (*p* = 0.0393; χ^2^ = 6.4871), as shown in Table 2. However, after running the data in a weighted jackknife procedure, the results showed no statistically significant difference (Pr > F = 0.0668).

### Patient Provided Access to EHR

Patients who reported that their providers maintained an EMR were used to determine how many patients were offered access to their medical records. Between 2017 and 2020 there was a steady increase, in both regions, in the number of patients who reported being offered access to their patient portal, but data are not shown. However, the two regions do not show a statistically significant difference (*p* = 0.1650; χ^2^ = 3.6031), which was confirmed with the weighted jackknife procedure, as shown in Table 2.

### Patient Use of EHR

Patients who were offered access to patient portals were used to determine the use of patient portals. Data show a statistically significant difference in the number of people who report using their patient portals between the two regions (*p* = 0.0097; χ^2^ = 6.6938), as shown in Table 2. Again, the weighted jackknife procedure showed no statistically significant difference between groups (Pr > F = 0.4658). However, the number of responses was low in the Appalachian Region, and data should be interpreted cautiously.

Due to variations between regions in race and education level, education and race were controlled to look at differences in patient portal use. Controlling for education and race did not show any additional statistically significant difference.

### Patient-reported Barriers for Non-Use of Patient Portals

The most cited reason for the non-use of patient portals is that patients prefer to speak directly to their provider. Both regions, across all iterations, cited this as the most common reason for non-portal use (79%). The second most common reason for both regions was that the patients did not need to use their patient portal within the last twelve months (52%–53%).

Another analysis was conducted to look at the number of times that respondents reported visiting a doctor/nurse/health professional within the last 12 months (variable: FreqGoProvider) compared to the response of “did not have a need to use their patient portal.” Of those patients who responded they did not have a need to use their patient portal within the last 12 months, 89% of those in the Appalachian Region and 87% in the surrounding U.S. Census region had seen a healthcare provider within the last 12 months. Additionally, between 21% and 24% (U.S. Census Region v. Appalachia) of those who reported not needing to use their patient portals had visited a provider five or more times within the last 12 months, data not shown.

## DISCUSSION

Through HIPAA, the HITECH Act, and the 21^st^ Century Cures Act, there has been an increased push to offer patients access to their health information and facilitate involvement in their own care.^11^,^20^ Meanwhile, research supports that involving patients in their care can improve health outcomes.^7^

Those living in the Appalachian Region have a higher risk of death due to heart disease (17% higher), cancer (10% higher), COPD (27% higher), stroke (14% higher), and diabetes (11% higher) compared to the rest of the U.S.^5^ These health disparities are further complicated by socioeconomic factors, as residents of rural areas are more likely to have lower incomes, have less than a high school education, be unemployed, be uninsured, and have less access to care.^5^,^21^ For those living in the Appalachian Region, increased use of patient portals could be one way to begin to bridge this gap in health outcomes.

While the study did not show a statistically significant difference using the weighted jackknife procedure in the use of patient portals overall, without the weighted data, there was a statistically significant difference between regions for their use. This is an important finding and should be further studied. Healthcare providers must understand the reasons behind the non-use of patient portals to increase patient portal use.

### Reasons for Non-Use of Portals

The study did not show a statistically significant difference between the two regions for reasons of non-portal use using the weighted jackknife procedure. However, two interesting themes were noted in the study for the non-use of patient portals for both regions. These two reasons offer some insight into patients’ possible lack of knowledge and education regarding the importance and benefits of patient portals:

#### Prefer to speak to the provider

Most patients reported that a reason for the nonuse of the portal was that they preferred to speak directly to the provider. This raises the question of patients' knowledge about the patient portal's purpose and benefits.

Patient portals provide patients with a secure electronic connection to the information contained in their medical records, including medications, immunizations, lab results, and health summaries.^22^ Patients can track their data over time to identify changes in their health. Portals can also allow patients to schedule/cancel appointments, request prescription refills, make payments, complete check-in forms, and view educational materials.^22^ Moreover, patient portals can send reminders for appointments and to schedule preventive care.

#### No need to use the portal

The second most cited reason for the non-use of portals is that patients do not feel a need to use the portal. As stated above, 87%–89% of patients who saw a healthcare provider within the last 12 months felt they did not need to use their portal.

Both results highlight the importance of educating patients on the purpose and benefits of using their patient portal. Patients who received training on using their patient portals were more likely to use them.^12^,^23^ Healthcare providers and the developers of patient portals should consider the ways to increase patient engagement based on data from this study. It is important to remember that knowledge alone will not necessarily change patient attitudes or behaviors. However, proper knowledge can help patients to understand the benefits of using patient portals.^24^

## IMPLICATIONS

### Recommendations for Future Research

This preliminary study uncovered differences in patient portal use in the Appalachian Region as well as factors that may be barriers to portal engagement more broadly. Yet further research is needed to understand better the access and use of patient portals in Appalachia.

A more in-depth analysis of the data should be conducted to examine other factors that impact the non-use of portals between the two regions. Regression models should be developed to investigate the extent to which certain factors play a role in the use of patient portals. Additionally, based on previous research, there is a need to look at the impact of income, rural area, and insurance status and their impact on portal use between the two regions.

A study tailored to patient portal use should be developed to examine use of patient portals in the Appalachian Region. This would allow researchers to look for variations in race, age, education, income, and insurance between regions within Appalachia, helping to target efforts to improve portal use. Further research is needed to expand upon questions about the non-use of patient portals and to learn more about why the adoption of patient portals is low.

### Limitations

There are a few limitations of this study. One limitation is the small sample size for the Appalachian Region. The number of responses in the dataset was 960 for all years combined (2017–2020), averaging about 250 responses per year. HINTS over samples high-minority areas, and the Appalachian Region has lower racial/ethnic diversity than in other areas, which could be one contributing factor to the smaller sample size. Additionally, the questions regarding patient portals in the HINTS dataset are limited. Therefore, an in-depth analysis of causal factors was difficult to examine.

While there are limitations to the dataset, the data provides a solid foundation to explore initial differences in patient portal use in the Appalachian Region. As stated, increasing the use of and engagement in patient portals could be one step toward decreasing health disparities for those living in Appalachia.

**SUMMARY BOX**

**What is already known about this topic?**

There has been research on the use of patient portals but none focusing specifically on the use of patient portals in Appalachia.

**What is added by this report?**

This report begins to look at how patients living in Appalachia access and use their patient portals.

**What are the implications for future research?**

Knowing that non-Hispanic whites in the Appalachian Region use their patient portals less than those in the surrounding U.S. Census regions can help healthcare professionals identify ways to encourage access and use of portals to improve the health of those living in the Appalachian Region.

## Figures and Tables

**Table 1:** Characteristics of Respondents in the Appalachian Region and U.S. Census Regions Surrounding Appalachia – NCI HINTS 5 Cycles 1–4 (2017–2020)

	Appalachian Region					U.S. Census Regions				
Year	All	2017	2018	2019	2020	All	2017	2018	2019	2020
*n*	*960*	*208*	*208*	*316*	*228*	*7388*	*1513*	*1639*	*2463*	*1773*
**Age**										
	57.9 (±16)	56.6 (±15.4)	58.3 (±15.9)	58.5 (±16.4)	58.1 (±16.0)	57.3 (±16.6)	56.9 (±16.2)	57.0 (±16.5)	57.6 (±16.8)	57.48 (±16.8)
**Gender**										
*Female (=2)*	504 (52.5%)	118 (56.7%)	94 (45.2%)	165 (52.2%)	127 (55.7%)	4040 (54.7%)	841 (55.6%)	924 (56.4%)	1300 (52.8%)	975 (55%)
*Missing (= −9/−7)*	71 (7.2%)	8 (3.9%)	17 (8.2%)	28 (8.9%)	18 (7.9%)	510 (6.9%)	75 (5.0%)	111 (6.8%)	199 (8.1%)	125 (7.1%)
**Race**										
*White (=2)*	681 (70.9%)	135 (64.9%)	141 (67.8%)	231 (73.1%)	174 (76.3%)	4174 (56.5%)	895 (59.2%)	936 (57.1%)	1375 (55.8%)	968 (54.6%)
*Black (=3)*	108 (11.3%)	32 (15.4%)	30 (14.4%)	27 (8.5%)	19 (8.3%)	1327 (18%)	269 (17.8%)	286 (17.5%)	452 (18.4%)	320 (18.1%)
*Hispanic (=1)*	30 (3.1%)	10 (4.8 %)	2 (0.9%)	11 (3.5%)	7 (3.1%)	694 (9.4%)	128 (8.5%)	141 (8.6%)	225 (9.1%)	200 (11.3%)
*Asian (=5)*	11 (1.2%)	5 (2.4%)	2 (0.9%)	2 (0.6%)	2 (0.9%)	260 (3.5%)	47 (3.1%)	62 (3.8%)	86 (3.5%)	65 (3.7%)
*Other (=4, 6, 7)*	27 (2.8%)	6 (2.9%)	8 (3.9%)	9 (2.9%)	4 (1.8%)	202 (2.7%)	43 (2.8%)	54 (3.3%)	62 (2.5%)	43 (2.4%)
*Missing (= −9)*	103 (10.7%)	20 (9.6%)	25 (12.0%)	36 (11.4%)	22 (9.7%)	731 (9.9%)	131 (8.7%)	160 (9.8%)	263 (10.7%)	177 (9.9%)
**Highest Level of Education**										
*Less than HS (=1)*	73 (7.6%)	10 (4.8%)	19 (9.1%)	30 (9.5%)	14 (6.1%)	475 (6.4%)	102 (6.7%)	119 (7.3%)	137 (5.6%)	117 (6.6%)
*HS (=2)*	228 (23.8%)	56 (26.9%)	49 (23.6%)	74 (23.4%)	49 (21.5%)	1384 (18.7%)	288 (19.0%)	307 (18.7%)	462 (18.8%)	327 (18.4%)
*Some college (=3)*	280 (29.2%)	71 (34.1%)	66 (31.7%)	78 (24.7%)	65 (28.5%)	2107 (28.5%)	427 (28.2%)	480 (29.3%)	716 (29.1%)	484 (27.3%)
*Bachelor's degree (=4)*	226 (23.5%)	37 (17.8%)	41 (19.7%)	90 (28.5%)	58 (25.5%)	1914 (25.9%)	395 (26.1%)	426 (26%)	643 (26.1%)	450 (25.4%)
*Postgraduate degree (=5)*	140 (14.6%)	33 (15.9%)	31 (14.9%)	41 (13%)	35 (15.4%)	1400 (18.9%)	291 (19.2%)	295 (18.0%)	474 (19.2%)	340 (19.2%)
*Missing (= −9/−7)*	13 (1.4%)	1 (0.5%)	2 (0.9%)	3 (1%)	7 (3.1%)	108 (1.5%)	10 (0.7%)	12 (0.7%)	31 (1.3%)	55 (3.1%)
**Marital Status**										
*Married (=1)*	464 (48.3%)	94 (45.2%)	103 (49.5%)	145 (45.9%)	122 (53.5%)	3520 (47.6%)	777 (51.4%)	774 (47.2%)	1140 (46.3%)	829 (46.8%)
*Living as Married (=2)*	36 (3.8%)	6 (2.9%)	3 (1.4%)	18 (5.7%)	9 (3.9%)	273 (3.7%)	46 (3.0%)	38 (2.3%)	113 (4.6%)	76 (4.3%)
*Divorced (=3)*	174 (18.1%)	38 (18.3%)	38 (18.3%)	62 (19.6%)	36 (15.8%)	1135 (15.4%)	212 (14.0%)	262 (16.0%)	385 (15.6%)	276 (15.6%)
*Widowed (=4)*	127 (13.2%)	26 (12.5%)	29 (13.9%)	48 (15.2%)	24 (10.5%)	843 (11.4%)	162 (10.7%)	213 (13.0%)	277 (11.3%)	191 (10.8%)
*Separated (=5)*	23 (2.4%)	5 (2.4%)	3 (1.4%)	9 (2.9%)	6 (2.6%)	203 (2.8%)	48 (3.2%)	45 (2.8%)	62 (2.5%)	48 (2.7%)
*Single, Never Married (=6)*	122 (12.7%)	36 (17.3%)	31 (14.9%)	31 (9.8%)	24 (10.5%)	1305 (17.7%)	254 (16.8%)	293 (17.9%)	453 (18.4%)	305 (17.2%)
*Missing (= −9/−7/−5)*	14 (1.5%)	3 (1.4%)	1 (0.5%)	3 (1%)	7 (3.1%)	109 (1.5%)	14 (0.9%)	14 (0.9%)	33 (1.3%)	48 (2.7%)

**Table 21:** Bivariate analysis of patient portal access and use within the last 12 months. The Appalachian Region v. U.S. Census regions surrounding Appalachia – NCI HINTS 5 Cycles 1–4 (2017–2020)

	Appalachian Region, *n (%)*	Surrounding U.S. Census regions, *n (%)*	*p*-value (chi-square)
**Provider Maintains EHR?**			
*Yes*	753 (79.01%)	5999 (82.28%)	*p* = 0.0393 (χ^2^ = 6.4708)
*No*	24 (2.52%)	175 (2.40%)	Weighted Pr > F = 0.0668 (χ^2^ = 5.4871)
*Unsure*	176 (18.47%)	1117 (15.32%)	
**Patient Offered Access to EHR?**			
*Yes*	471 (62.88%)	3908 (65.52%)	*p* = 0.1292 (χ^2^ = 4.0932)
*No*	203 (27.10%)	1580 (26.49%)	Weighted Pr > F = 0.9790 (χ^2^ = 0.0424)
*Unsure*	75 (10.01%)	477 (8.00%)	
**Patient Portal Use:** Accessed patient portal within previous 12 months?			
*Yes*	268 (57.14%)	2459 (63.26%)	*p* = 0.0097 (χ^2^ = 6.6938)
*No*	201 (42.86%)	1428 (36.74%)	Weighted Pr > F = 0.5339 (χ^2^ = 0.4658)

## Footnotes

1 Cycle 4 (Updated May 2022); Cycle 3 (Updated April 2021); Cycle 2 (Oct 2020); Cycle 1 (June 2020)

## References

[1] Martin AB Hartman M Lassman D Catlin A National health care spending 2019: Steady growth for the fourth consecutive year Health Aff 2021 40 1 14 24 10.1377/hlthaff.2020.02022 33326300

[2] Centers for Medicare and Medicaid Services [CMS] 2020 National health expenditure data: Historical Available at: https://www.cms.gov/Research-Statistics-Data-and-Systems/Statistics-Trends-and-Reports/NationalHealthExpendData/NationalHealthAccountsHistorical

[3] Buttorff C Ruder T Bauman M Multiple chronic conditions in the United States Santa Monica CA Rand Corp 2017 Available at: https://www.rand.org/content/dam/rand/pubs/tools/TL200/TL221/RAND_TL221.pdf

[4] Holman HR The relation of the chronic disease epidemic to the health care crisis ACR Open Rheumatol 2020 2 3 167 73 10.1002/acr2.11114 32073759 PMC7077778

[5] Appalachian Regional Commission [ARC] The Appalachian Region: A data overview from the 2014–2018 American Community Survey Washington DC ARC 2020 Available at: https://www.arc.gov/report/theappalachian-region-a-data-overview-from-the-2014-2018-americancommunity-survey/ Accessed Aug. 9, 2023

[6] Health Resources & Services Administration [HRSA] Health Literacy 2019 Available at: https://www.hrsa.gov/about/organization/bureaus/ohe/healthliteracy/index.html#:~:text=Health%20literacy%20is%20the%20degreeOlder%20adults

[7] Committee on Quality of Health Care in America Crossing the quality chasm: A new health system for the 21st Century Washington DC Institute of Medicine 2001 10.17226/10027

[8] Kruse CS Argueta DA Lopez L Nair A Patient and provider attitudes towards the use of patient portals for the management of chronic disease: A systematic review J Med Internet Res 2015 17 2 10.2196/jmir.3703 PMC4376181 25707035

[9] Ancker JS Barron Y Rockoff ML Hauser D Pichardo M Szerencsy A Calman N Use of an electronic patient portal among disadvantaged populations J Gen Intern Med 2011 26 10 1117 23 10.1007/s11606-011-1749-y 21647748 PMC3181304

[10] Anthony DL Campos-Castille C Lim PS Who isn’t using patient portals and why? Evidence and implications from a national sample of US adults Health Aff 2018 37 12 1948 54 10.1377/hlthaff.2018.05117 30633673

[11] El-Toukhy S Mendez A Collins S Perez-Stable EJ Barriers to patient portal access and use: Evidence from the Health Information National Trends Survey J Am Board Fam Med 2020 33 6 953 68 10.3122/jabfm.2020.06.190402 33219074 PMC7849369

[12] Antonio MG Petrovskaya O Lau F The state of evidence in patient portals: Umbrella Review J Med Internet Res 2020 22 11 10.2196/23851 PMC7688386 33174851

[13] Graetz I Gordon N Fung V Hamity C Reed ME The digital divide and patient portals: Internet access explained differences in patient portal use for secure messaging by age, race, and income Med Care 2016 54 8 772 9 10.1097/MLR.0000000000000560 27314262

[14] Otokiti A Williams KS Warsame L Impact of digital divide on the adoption of online patient portals for self-motivated patients Healthcare Informatics Res 2020 26 3 220 8 10.4258/hir.2020.26.3.220 PMC7438699 32819040

[15] US Department of Health and Human Services America’s health literacy: Why we need accessible health information 2008 Available at: https://www.ahrq.gov/sites/default/files/wysiwyg/health-literacy/dhhs-2008-issue-brief.pdf

[16] Irizarry T Shoemake J Nilsen ML Czaja S Beach S Dabbs AD Patient portals as a tool for health care engagement: A mixed-method study of older adults with varying levels of health literacy and prior patient portal use J Med Internet Res 2017 19 3 e99 10.2196/kmir.7099 28360022 PMC5391436

[17] Wijesundara JG Fukunaga MI Ogarek J Barton B Fisher L Preusse P Electronic health record portal messages and interactive voice response calls to improve rates of early seasonal influenza vaccination: Randomized controlled trial J Med Internet Res 2020 22 9 10.2196/16373 PMC7547389 32975529

[18] Sequist TD Zaslavsky AM Colditz GA Ayanian JZ Electronic patient messages to promote colorectal cancer screening: a randomized controlled trial Arch Intern Med 2010 171 7 636 41 10.1001/archinternmed.2010.467 21149743 PMC3169179

[19] Dharod A Bellinger C Foley K Case LD Miller D The reach and feasibility of an interactive lung cancer screening decision aid delivered by patient portal Appl Clin Inform 2019 10 01 19 27 10.1055/s-0038-1676807 30625501 PMC8438623

[20] US Department of Health and Human Services Individuals’ rights under HIPAA to access their health information 45 CFR § 164, 524 Available at: https://www.hhs.gov/hipaa/forprofessionals/privacy/guidance/access/index.html

[21] Wheeler SB Davis MM “Taking the bull by the horns”: Four principles to align public health, primary care, and community efforts to improve rural cancer control J Rural Health 2017 33 4 345 9 10.111/jrh.12263 28905432 PMC5824432

[22] HealthIT.gov What is a patient portal? 2017 Available at: https://www.healthit.gov/faq/what-patient-portal

[23] Grossman LV Masterson Creber RM Benda NC Wright D Vawdrey DK Ancker JS Interventions to increase patient portal use in vulnerable populations: a systematic review J Am Med Inform Assn 2019 28 8–9 855 70 10.1093/jamia/ocz023 PMC6696508 30958532

[24] DiClemente RJ Salazar LF Crosby RA Health behavior theory for public health: Principles, foundations, and applications Burlington MA Jones & Bartlett Learning 2019 978-0763797539

